# Molecular Mechanisms of Amylin Turnover, Misfolding and Toxicity in the Pancreas

**DOI:** 10.3390/molecules27031021

**Published:** 2022-02-02

**Authors:** Diti Chatterjee Bhowmick, Zhanar Kudaibergenova, Lydia Burnett, Aleksandar M. Jeremic

**Affiliations:** 1 Department of Molecular and Cellular Endocrinology, Diabetes and Metabolism Research Institute, City of Hope’s Beckman Research Institute, Duarte, CA 91010, USA; dcbhowmick@coh.org; 2 Department of Biological Sciences, The George Washington University, Washington, DC 20052, USA; zhannkud81@gwmail.gwu.edu (Z.K.); burnettlr@gwmail.gwu.edu (L.B.)

**Keywords:** human islet amyloid polypeptide, pancreas, transcription, secretion, aggregation, proteotoxicity, cholesterol, lipids, type-2 diabetes mellitus, islet amyloidosis

## Abstract

Amyloidosis is a common pathological event in which proteins self-assemble into misfolded soluble and insoluble molecular forms, oligomers and fibrils that are often toxic to cells. Notably, aggregation-prone human islet amyloid polypeptide (hIAPP), or amylin, is a pancreatic hormone linked to islet β-cells demise in diabetics. The unifying mechanism by which amyloid proteins, including hIAPP, aggregate and kill cells is still matter of debate. The pathology of type-2 diabetes mellitus (T2DM) is characterized by extracellular and intracellular accumulation of toxic hIAPP species, soluble oligomers and insoluble fibrils in pancreatic human islets, eventually leading to loss of β-cell mass. This review focuses on molecular, biochemical and cell-biology studies exploring molecular mechanisms of hIAPP synthesis, trafficking and degradation in the pancreas. In addition to hIAPP turnover, the dynamics and the mechanisms of IAPP–membrane interactions; hIAPP aggregation and toxicity in vitro and in situ; and the regulatory role of diabetic factors, such as lipids and cholesterol, in these processes are also discussed.

## 1. Introduction

Amyloid formation or amyloidogenesis, an intrinsic property of all polypeptides [1], is a process by which soluble proteins aggregate into insoluble structurally conserved unbranched fibers. These are characterized by resistance to proteinase K digestion, dye binding specificity and ordered β-sheet-rich structure [2]. Amyloids can be broadly categorized into functional and detrimental. Functional amyloids are an integral part of the normal physiology of the cell and include curli, chaplin, URE2p and PmeL17. Curli, found in *E. coli,* plays a role in biofilm formation and mediates infection. Chaplin, found in Streptomyces, plays a role in protection against water surface tension. URE2p, found in *S. Cerevisiae*, plays a role in nitrogen catabolism. Pmel17, found in humans, plays a role in melanin synthesis [3,4,5]. On the other hand, clinical and molecular studies have revealed a diverse group of detrimental amyloids, which cause protein misfolding in amyloid-driven diseases. These include huntingtin implicated in Huntington’s disease, α-synuclein implicated in Parkinson’s disease, prion protein implicated in Creutzfield–Jacob’s disease, superoxide dismutase implicated in amyotrophic lateral sclerosis, amyloid-β (Aβ) peptide implicated in Alzheimer’s disease, transthyretin implicated in transthyretin familial amyloidosis and Tau implicated in frontotemporal lobar degeneration. Other notable examples are serum amyloid A linked with inflammation-linked amyloidosis; apolipoproteins implicated in systemic amyloidosis and atherosclerosis; and human pancreatic islet amyloid polypeptide (hIAPP), or amylin, a principal component and cause of islet amyloidosis. Moreover, hIAPP is a small 37 aa–long peptide hormone produced and co-secreted with insulin by pancreatic β-cells. Insulin and hIAPP act in a synergistic manner to regulate blood glucose levels and other important cellular functions [6,7].

The onset of T2DM is characterized by three determining factors: the insufficient ability of pancreatic β-cells to secrete insulin, decreased insulin sensitivity of peripheral tissues, and the deposition of hIAPP-derived aggregates, or amyloid [6,8,9,10]. During the last two decades, there has been significant progress in understanding the cytotoxic mechanism of human amylin oligomers and aggregates formed intracellularly, extracellularly or both [11,12,13,14,15,16,17,18,19]. Studies performed in primates strongly support the concept that islet amyloidosis and β-cell apoptosis are two key determinants of islet dysfunction [20,21]. However, the cellular processes regulating amylin turnover and hIAPP-evoked β-cell apoptosis in human islets remain poorly understood. Consequently, there exist no efficient treatments that prevent hIAPP aggregation and toxicity in humans. hIAPP toxicity is an important health issue. Over 90% of type-2 diabetic patients harbor the toxic amylin oligomers and aggregates (to variable degrees) that contribute to β-cell mass loss in T2DM [6,22,23]. In contrast to this large body of studies on mechanisms of hIAPP aggregation and toxicity in the pancreas, the molecular mechanisms that control hIAPP biosynthesis, recycling and interactions in normal and stressed pancreatic β-cells have been less explored. Dynamics and mechanisms of hIAPP intracellular synthesis, secretion and degradation (collectively referred to here as turnover) could be important for the prevention of hIAPP intracellular accumulation, aggregation and detoxification in β-cells. A better understanding of hIAPP turnover pathways and their relevance to islet amyloidosis may open new avenues for treatment of T2DM.

In view of this, here we summarize findings of cellular and reconstituted studies that explored roles of biosynthetic/degradation pathways and cellular constituents, such as cholesterol, lipids and biological metals in hIAPP turnover and aggregation in the pancreas that have important relevance for etiology of islet amyloidosis and T2DM. The clinical implications and molecular mechanisms of hIAPP–lipid and hIAPP–metal interactions in respect to its aggregation and toxicity in pancreas is also reviewed. In this review, we discuss an intriguing idea and evidence for possible crosstalk between hIAPP and other amyloid proteins and their possible relevance for brain and islet amyloidosis. Finally, advancements in small-molecule-amyloid-inhibitor therapy relying on natural products is also reviewed.

## 2. Transcriptional Mechanisms Implicated in IAPP Synthesis in Pancreatic Cells

**Figure 1:**
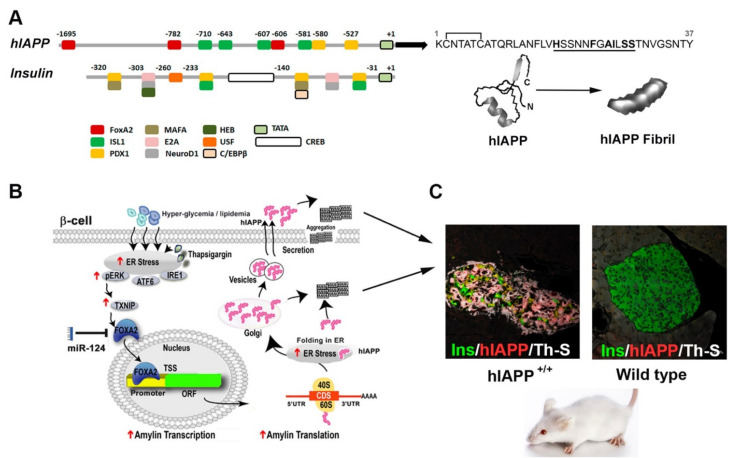
Molecular determinants of hIAPP synthesis, aggregation and toxicity in pancreatic islets. (**A**) Diagram depicts main transcriptional regulatory sites and factors from hIAPP and insulin promoters. Primary sequence of fully processed mature hIAPP form is shown on the right. Amyloidogenic region in hIAPP amino acids sequence is underlined. AFM micrograph of a single fibril self-assembled from mature synthetic hIAPP monomers is shown below. Adapted from Reference [46]. (**B**) Diagram depicts main steps in hIAPP synthesis in glucose-challenged or ER-stressed pancreatic β-cells, including activation of a central TXNIP/FOXA2-mediated signaling pathway. Following processing, hIAPP is stored together with insulin in secretory vesicles. Disproportionate production and/or processing of hIAPP in human islets may initiate its aggregation and consequently β-cell stress and islet amyloidosis. (**C**) Excessive intracellular and/or extracellular accumulation of protein aggregates in hIAPP transgenic mice induces a loss in β-cell mass and hyperglycemia, which are main pathological attributes of T2DM. Note a decrease in insulin levels (green) with simultaneous accumulation of hIAPP (red) and thioflavin T (ThT)-positive protein aggregates (white) in hIAPP transgenic mouse islets as compared to wild-type mice islets which are hIAPP- and aggregate-free. Additionally, note severe islet cells atrophy and distortion of hIAPP transgenic mouse islets as compared to morphologically and functionally preserved islets from non-diabetic wild-type mice. Confocal micrographs adapted from Reference [33].

The two major glucose-regulating pancreatic hormones are hIAPP and insulin [6,24,25]. The hormone hIAPP is encoded by one single-copy gene in chromosome 12 and contains three exons [26,27,28], of which the last two exons encode the full preproIAPP molecule (89 aa) [26,27,28]. Human insulin is encoded by the insulin gene (*INS*) located on chromosome 11p15.5 and consists of several exons [29]. Similarly, IAPP insulin is initially synthesized as preproinsulin. Through the process of co-translational insertion into the lumen of the ER and the cleavage of the N terminal 24 aa signal sequence, both preproIAPP and preproinsulin are converted to proIAPP and proinsulin, respectively [6,30,31] (Figure 1A). Both proIAPP and proinsulin undergo similar post-translational processing by prohormone convertases PC2 and PC1/3, as well as carboxypeptidase E [6]. The mature 37 aa–long hIAPP form adopts a soluble, predominately random coil conformation and is co-released twith insulin from islet β-cells (Figure 1A,B). However, under certain pathophysiological (glucolipotoxic and/or ER stress) conditions, IAPP biosynthesis increases several folds [32], which may lead to its misfolding and intracellular and/or extracellular accumulation in the form of soluble toxic oligomers and/or insoluble fibrils (Figure 1B,C). Notably, β-cell-specific expression of hIAPP under the insulin promoter stimulates islet amyloidosis, glucose imbalance, ASK-1-mediated apoptosis of β-cells and, ultimately, diabetes in transgenic mice (Figure 1C) [33].

Regardless of similar gene structure and post-translation processing mechanisms, these two master regulators of glucose homeostasis differ significantly from each other with respect to their levels of gene and protein expression and their responsiveness to a glucose stimulus. For example, a study by Mulder et al. (1996) showed that, in rat pancreatic islets, the steady-state level of IAPP mRNA is 10-fold less than the steady-state insulin mRNA levels [34]. Interestingly, microarray and gene expression analysis revealed that 24 h of high glucose-stimulated a ~10 fold increase in IAPP’s mRNA where the insulin transcript levels remained unaltered [35]. Based on this knowledge, it has been proposed that post-transcriptional modifications and differential promoter regulation could account for this differential expression of insulin and IAPP [36,37]. It has been demonstrated that hIAPP gene transcription is regulated by a compound promoter region spanning from −2798 to + 450 (with respect to the transcription start site) [38] and both hIAPP and insulin promoters share common transcription factors such as PDX1 and ISL1 [38,39,40,41,42] (Figure 1A). However, despite this structural similarity, ISL1 is involved in the regulation of IAPP but not insulin promoter activity, whereas glucose regulation of both IAPP and insulin promoter requires binding of transcription factor PDX1 [37,39,40,43]. To add to this, in contrast to insulin, the IAPP promoter includes three FoxA2 binding sites among which the proximal FoxA2 binding site (−606 bp) is highly conserved across species [37]. FoxA2 is a member of the hepatocyte nuclear factor 3 or FoxA family of proteins and bind to DNA via its centrally located DNA-binding forkhead box domain [44]. Moreover, hIAPP but not insulin promoter activity requires additional calcium responsive elements that are yet to be identified [39,45].

A study by Jing et al. (2014) demonstrated that, in the β-cells, the major glucose-induced gene, a thioredoxin-interacting protein (TXNIP), stimulated IAPP transcription by increasing the expression of transcription factor FoxA2, thereby revealing a novel β-cell-signaling cascade under physiological conditions [37] (Figure 1B). Intriguingly, in contrast to IAPP, TXNIP downregulated insulin production in pancreatic β-cells [47]. Based on these results, it can be predicted that the differential regulation of IAPP and insulin transcription could be accounted at least in part by the involvement of the FoxA2 promoter element. Independent studies demonstrated that ER-stress, an important diabetogenic factor, augments TXNIP production in pancreatic β-cells (Figure 1B) [48,49]. Similar to this, induction of ER stress in primary human islets stimulated a major increase in hIAPP mRNA and protein levels, as well as increased IAPP promoter activity (Figure 1B) [32]. Reduced IAPP promoter activity in the ER-stressed β-cells transiently expressing FoxA2 dominant-negative (DN) construct further established the important contribution of FoxA2 and transcriptional mechanisms in IAPP expression under pathophysiological-relevant conditions (Figure 1B) [32]. These studies raised the important questions whether and to which extent high glucose-stimulated hIAPP expression is ER-stress dependent? Clearly, efforts to reduce hIAPP overproduction and potentially aggregation, in ER-stressed or high glucose-challenged β-cells are and will continue to be of high clinical value. The current studies demonstrate that chronic HG treatment may concurrently induce ER stress and hIAPP transcription [32], thus supporting this concept. Comparative transcriptomic studies revealed that the hIAPP mRNA expression pattern in ER-stressed β-cells strongly correlated with TXNIP transcript levels [32]. Similarly, a seminal study by Shalev and colleagues demonstrated the important regulatory role of TXNIP in FoxA2-mediated IAPP expression in normal and HG-treated islet β-cells [37]. Thus, studies support the idea that high glucose and ER-stress may stimulate hIAPP production in β-cells via a shared TXNIP/FoxA2-signaling pathway (Figure 1B). In line with this concept, CHIP studies demonstrated that the binding of carbohydrate response element-binding protein (ChREBP) to the *txnip* promoter is enhanced in high-glucose and ER-stressed β-cells, thereby augmenting TXNIP transcription under these adverse conditions [47,48,49]. However, it remains to be clarified the extent to which TXNIP-mediated metabolic and stress pathways overlap and drive HG-induced hIAPP synthesis, and equally important how they may be relevant for amylin’s aggregation and toxicity.

**Figure 2:**
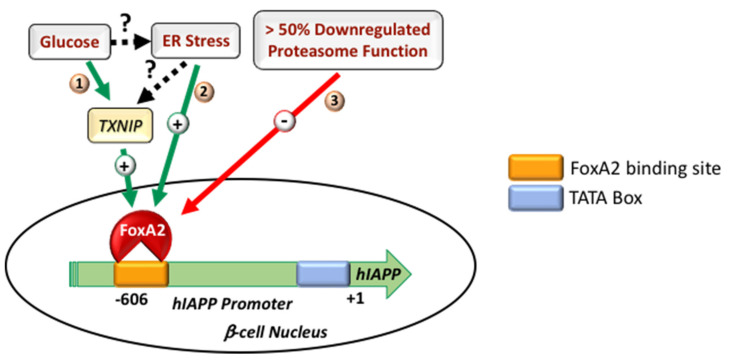
Regulatory mechanisms driving IAPP transcription in normal and stressed pancreatic β-cells. (**1**) High glucose induces expression of the major glucose regulated gene TXNIP, which, in turn, upregulates IAPP transcription by increasing the expression and promoter binding of IAPP specific transcription factor FoxA2 in the β-cell. (**2**) Similarly, ER stress upregulates IAPP transcription in β-cells by increasing IAPP promoter’s occupancy for transcription factor FoxA2, for which the binding of could be TXNIP-dependent or independent. (**3**) Severe inhibition of proteasome function and associated protein stress downregulate IAPP transcription by attenuating FoxA2 binding at the IAPP promoter in the β-cell. Green arrows depict positive regulatory pathways, and red arrow depicts an inhibitory pathway. Different signaling branches are numbered. Dashed arrows depict hypothetical signaling branches.

In addition to biosynthetic pathways, studies suggest an important contribution of degradation (proteolytic) pathways to IAPP transcription in the pancreas. For instance, impaired activity/function of the ubiquitin–proteasome system (UPS) in human islet β-cells, elicited by diabetogenic stress conditions, resulted in downregulated IAPP promoter activity and decreased IAPP mRNA and protein levels [50]. FoxA2 binding at the IAPP promoter region was proposed as a limiting factor for IAPP transcription in proteasome-impaired rodent β-cells [50] (Figure 2). These results indicate the existence of a complex regulatory network of IAPP production in the stressed β-cell (Figure 2). Accordingly, although proteasome function is required for the full expression of IAPP’s crucial transcription factor FoxA2, stress conditions such as high glucose alone do not significantly change the protein level of FoxA2 in the β-cell [50]. Hence, promoter occupancy, but not the steady-state level of at least FoxA2, appears to be a crucial and curbing factor in IAPP transcription in the β-cell under various pathophysiological conditions (Figure 1B and Figure 2). It has also been reported that TXNIP is an important regulator of microRNA (miRNA) expression in the β-cell [37,47]. MicroRNAs (miRNAs) represent the small ~22 nucleotides non-coding regulatory RNA pool that regulates gene expressions via binding to the 3′-UTR of target mRNAs [51,52,53]. In addition to the regulatory role of the proteasome on FoxA2 expression [50], the study also revealed miR-124a-mediated regulation of FoxA2 expression in the β-cell which is, again, regulated upstream via TXNIP [37]. Hence, the TXNIP/miR-124a/FoxA2/IAPP-signaling cascade represents a novel signaling branch in the β-cell that could be co-regulated by various stress factors related to diabetes (Figure 1B and Figure 2). In addition to miR-124a, Chr 14q32 miRNAs, namely miR-376a and miR-432, had also been implicated in the regulation of IAPP expression in the β-cell [54]. Marked downregulation of the Chr 14q32 locus miRNAs in the T2DM donor islets indicates a strong correlation between dysregulated IAPP expression and progression of diabetes pathogenesis [54], the mechanistic details of which need further experimental clarification.

## 3. Proteolytic Pathways Regulate IAPP (Mis)Folding, Synthesis and Degradation

Increasing evidence suggests the contribution of hIAPP-derived toxic oligomers and aggregates toward the progressive loss of functional β-cell mass during T2DM [6,24,25,55,56]. Bioinformatics and mutational studies confirmed that the 18–29 aa segment of mature hIAPP is the primary amyloidogenic segment [57,58,59] (Figure 1A). Interestingly, despite the presence of the high-sequence homology between human and rat IAPP (rIAPP) proteins, the differences of a few key amino acids in the amyloidogenic region of the IAPP (18–29 aa) could significantly change IAPP’s aggregation and cytotoxic potential. For example, compared to hIAPP, an absence of histidine and the presence of proline in the amyloidogenic region (18–29 aa) of rIAPP has been shown to prevent its aggregation and toxicity in vitro and in rodents [19,60,61]. In addition to these, in vitro studies showed increased aggregation potential of S20G hIAPP in solution, demonstrating the regulatory role of other polar amino acids from the amyloidogenic region of hIAPP in aggregation and islet amyloid formation [62]. In support of this, a subset of Chinese and Japanese populations harboring the S20G mutation in mature hIAPP showed an increased risk of developing T2DM.

In addition to its 18–29 aa amyloidogenic segment, various IAPP molecular conformations seems to be important for its aggregation and toxicity. It has been suggested that pre-fibrillar low-MW oligomeric species, rather than the mature fibrillar forms of hIAPP, induced membrane damage and β-cell death [13,63,64,65,66,67]. Similarly, impaired turnover and cellular processing of hIAPP contribute significantly toward the progressive β-cell failure during T2DM [6,25]. In addition to the above-described transcriptional mechanisms, recent studies suggest that the proteolytic ubiquitin–proteasome system (UPS) plays an important role in controlling the intracellular levels of hIAPP in primary human islets [50,68]. The proteasome is a multi-subunit catalytic compartment of cells and is primarily known for the degradation of cytosolic and nuclear proteins, as well as for regulation of gene transcription in eukaryotes [69,70]. Studies using proteasome proteolytic inhibitors, lactacystin and epoxomicin revealed increased hIAPP content and toxicity in hIAPP-treated clonal β-cells [71]. Complementary studies showed stimulation of the proteasome function with protein activator 28 (PA28) and diminished cytotoxic effects of hIAPP, indicating contribution of proteasome in degradation of exogenous hIAPP [71,72]. A sequel study showed a similar fate for endogenous hIAPP as proteasome complex also regulated biosynthesis and degradation of hIAPP in human islets [50]. Interestingly, a moderate inhibition of proteasome’s function (40% or less) raised the intracellular hIAPP levels without significantly altering hIAPP’s gene expression and secretion, indicating proteasome-mediated degradation of hIAPP [50]. Intriguingly, a more severe impairment of the proteasome’s proteolytic function (>50%) negatively regulates hIAPP transcription and secretion (Figure 2) [50]. Inhibition of proteasome function also downregulated transcript levels of IAPP key transcription factor FOXA2, although FOXA2 protein levels start to decline with a delay of ~6 h following proteasome function inhibition [50]. Consistent with this finding, a promoter activity study demonstrated efficient blockade of IAPP promoter activity following long-term (>6 h), not brief (≤6 h), proteasome inhibition in the beta-cell [50], suggesting FOXA2-dependent and -independent mechanisms of IAPP synthesis in proteasome-impaired islet beta-cells [50]. In agreement with a FoxA2-dependent mechanism, ChIP studies revealed that promoter occupancy of FoxA2 at the rat IAPP promoter region is an important and limiting factor for IAPP expression in proteasome-impaired murine cells [50] (Figure 2).

**Figure 3:**
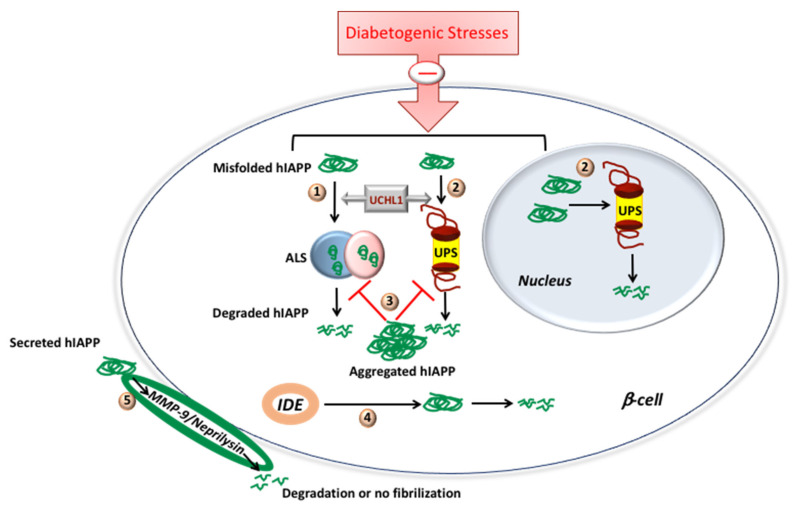
Proteolytic pathways involved in IAPP degradation. (**1**) ALS is involved in degradation of misfolded IAPP localized outside nucleus. (**2**) UPS regulates degradation of misfolded IAPP localized in cytosol and nucleus. Diabetogenic stresses downregulate both (**1**) and (**2**). UCHL1 probably regulates both (**1**–**3**) hIAPP aggregate inhibits both (**1**) and (**2**). (**4**) IDE degrades cytosolic hIAPP aggregates. (**5**) MMP-9 and neprilysin degrade secreted form of hIAPP and/or inhibits fibril formation, thereby preventing its aggregation on the beta-cell.

Another important question besides the role of proteasome on hIAPP degradation is whether hIAPP may modulate proteasome’s proteolytic function in the beta-cell. Casas et al. (2007), using mouse clonal beta-cell MIN6 and primary human islets, showed that hIAPP, but not rIAPP, treatment leads to a significant decrease of proteasomal activity [72] (Figure 3). In partial agreement, using RIN-m5F cells (rat clonal beta cell line), Singh et al. (2016) demonstrated the inhibitory effects of both hIAPP and rIAPP on proteasomal activity [71] (Figure 3). On the other hand, adenovirus-mediated overexpression of rIAPP or hIAPP in primary human islets resulted in a comparable increase of proteasomal activity compared to non-transduced control cells [68]. By contrast, islets of hIAPP transgenic rats and wild-type rats failed to show any differential proteasomal activity [68]. Studies showed both stimulatory and inhibitory effects of IAPP isoforms on proteasomal activity: differential cellular background and model systems, different methods of preparation of synthetic IAPPs and different extents of IAPP overexpression could account for these discrepancies. Studies have also reported the downregulated proteasome activity, accumulation of polyubiquitinated proteins [27,50,73] and deficiency in the deubiquitinating enzyme UCHL1 (ubiquitin carboxyl-terminal esterase L1 [ubiquitin thiolesterase]) [73] in the T2DM donor islets, suggesting a dysfunctional ubiquitin/proteasome system (UPS) (Figure 3).

The other important cellular degradation system besides the ubiquitin/proteasome system is the autophagy/lysosomal system (ALS). Costes and colleagues, using mouse genetics, showed that UCHL1 deficit/dysfunction resulted in increased β-cell apoptosis by increased accumulation of autophagosomes, SQSTM1/p62-positive cytoplasmic inclusions and polyubiquitinated proteins with lysine 63-specific ubiquitin chains in hIAPP transgenic mice, indicating a defect in the autophagy/lysosomal pathway [73] (Figure 3). Importantly, both in vitro and in vivo studies provided compelling evidence for the role of the autophagy/lysosomal pathway in IAPP clearance in β-cells, an action which is independent of the confounding effect of hyperglycemia [50,74,75,76]. Specifically, attenuation and stimulation of the lysosomal degradation pathway sensitized and protected β-cells to hIAPP-induced cytotoxicity, respectively [74,75,76] (Figure 3). The details about crosstalk between ALS and IAPP have already been discussed in other reviews [45,77]. Taken together, not only ALS is important for proper IAPP turnover and folding in cells, but correct IAPP folding/function is important for the normal operation of proteolytic systems [77], indicating the existence of a multifaceted regulation between hIAPP and autophagy/lysosome systems in β-cells.

In addition to the UPS and ALS, studies have also revealed the important contribution of insulin degrading enzyme (IDE) in the degradation and clearance of synthetic IAPP in cell-free systems and immortalized cell lines [78,79,80] (Figure 3). Although IDE inhibition increases β-cell toxicity and apoptosis when synthetic hIAPP has been applied exogenously in vitro, IDE inhibition by its inhibitor Ii1 did not increases amyloid deposits in the hIAPP transgenic mice islets under the conditions that favor amyloid formation or β-cell loss [81]. In contrast to IDE, the inhibition of two other islet proteases, namely matrix metalloproteinase-9 (MMP-9) and neprilysin, increased islet amyloid formation and β-cell loss [82,83]. MMPs are extracellular gelatinases, and, although both MMP-9 and mRNA are present in mouse islets, only MMP-9 activity can be detected [82]. In the hIAPP transgenic mouse islets, both a broad-spectrum MMP inhibitor and an MMP-2/9 inhibitor augmented amyloid formation and β-cell death. Contrarily, using a specific MMP-2 inhibitor, the extent of amyloid formation and β-cell death was unchanged [82]. These results point toward the involvement of MMP-9 in regulating islet amyloid deposition and its toxic effects via degradation of hIAPP [82] (Figure 3). Similarly, inhibition or upregulation of neprilysin (a metallopeptidase), using a specific inhibitor/adenovirus encoding neprilysin showed >50% up- or downregulation of amyloid deposition and beta-cell apoptosis, respectively, indicating contribution of neprilysin in hIAPP degradation [83]. Interestingly, where mass spectrometric analysis using neprilysin incubated hIAPP failed to verify neprilysin-induced hIAPP cleavage, fluorescence-based thioflavin T binding studies and electron microscopy demonstrated reduced hIAPP fibrillogenesis [83]. These results all together point toward that neprilysin inhibition of fibril formation, but not hIAPP degradation [83], whereas MMP-9 reduces islet amyloid formation though increased hIAPP degradation [82]. In line with this idea, it has been proposed that different cellular localization of IDE (cytosolic) and MMP-9 (extracellular matrix), and, therefore, distinct accessibility to IAPP species (cytosolic vs. secreted), contribute to differential roles and effectiveness of IAPP degradation by these two proteolytic enzymes in β-cells [81].

Together, these data indicate the existence of complex and independent but not mutually exclusive proteolytic pathways that regulate hIAPP intracellular and extracellular levels, thereby protecting cells from amylin accumulation and aggregation. Thus, any dysregulation of proteolytic pathway(s) may potentially disrupt the fine balance between hIAPP turnover and global proteostasis in β-cells and accelerate the progression of diabetes and its pathogenesis (Figure 3).

## 4. Lipids and Cholesterol as Modulators of Amylin Folding and Aggregation

In addition to cell’s diverse proteolytic system, a myriad of reconstituted and cellular studies show that membrane-associated lipids and cholesterol act as potent regulators of hIAPP aggregation and toxicity [7]. Reconstituted spectroscopy and microscopy studies unveiled synthetic anionic lipids and cholesterol as accelerators and inhibitors of hIAPP aggregation in solution and on membranes, respectively [46,84,85]. In line with these reconstituted studies, hIAPP aggregates were discovered in close proximity to islet β-cells, and some fibrils integrated into the β-cell plasma membranes (PM) [86]. This finding implies that hIAPP-membrane interactions are essential for both hIAPP aggregation on the cell surface and for the integrity and function of the β-cell PM. Biophysical studies linked hIAPP toxicity to its ability to disrupt the fluidity and organization of cellular membranes [19,87,88]. Thus, understanding hIAPP–membrane interactions and amylin aggregation on the cell membrane are important for understanding the etiology of islet amyloidosis and T2DM.

Furthermore, hIAPP is a positively charged (cationic) peptide. Therefore, it is conceivable that it may interact with negative charged lipids from plasma and endo-membranes, such as phosphatidylserine (PS). This idea was first tested by Miranker and colleagues [85], and later confirmed by several independent biochemical studies, showing accelerated hIAPP aggregation and random coil to β-sheet conformational transition in solution in the presence of PS and other anionic lipids [46,84,89,90,91]. Specifically, negatively charged PS-containing liposomes potentiated hIAPP aggregation by increasing the rate of amylin fibrilization [46]. PS-enriched liposomes also shortened the lag phase, suggesting that electrostatic interactions between the peptide and the lipid accelerate nucleation, which is a rate-limiting step in hIAPP aggregation [88,92]. Interestingly, the inclusion of cholesterol, an essential component of cellular membranes, into anionic liposomes attenuated the stimulatory effect of PS on hIAPP aggregation in solution. The cholesterol exhibited a similar restricting (inhibitory) effect on hIAPP deposition across negatively charged planar membranes. Thermodynamic and kinetical calculations revealed that the presence of negatively charged liposomes decreases the activation energy (E_a_) of hIAPP aggregation by ΔE_a_ = −3.7 kJ/mol, which, in turn, increases the rate of its aggregation by ~4 folds [46]. Conversely, inclusion of cholesterol in anionic liposomes reversed their stimulatory effect on hIAPP aggregation in solution by increasing the activation energy by ΔE_a_ = 845 J/mol [46]. Importantly, despite the marked difference in hIAPP aggregation rates for three liposomal formulations, hIAPP fibrilization followed first-order kinetics [46], indicating that anionic lipids and cholesterol do not change the mechanism of hIAPP aggregation but rather act as catalyst and inhibitor, respectively. In support of this conclusion, inclusion of cholesterol decreases the rate of the conformational transition of hIAPP from random coil to β-sheets [46], a common and required structural step in aggregation of amyloid proteins [93].

**Figure 4:**
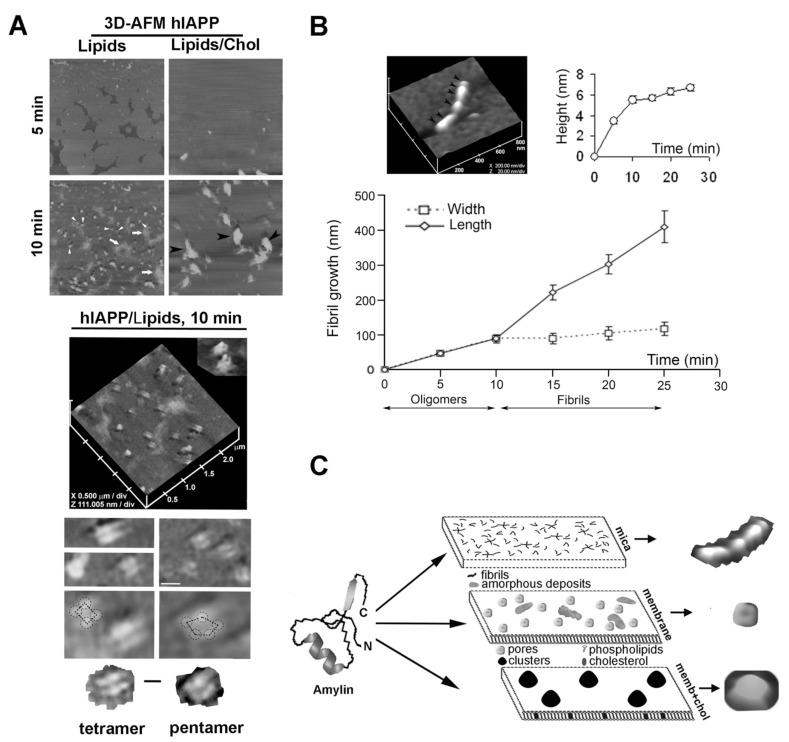
Polymerization pathways and polymorphic structures of hIAPP on different surfaces. (**A**) Time-lapse 3D-AFM analysis of hIAPP aggregation on soft negatively charged lipid/sterol planar membranes. Freshly dissolved monomeric hIAPP was added to preformed planar lipidic membranes of different composition, and the modulatory effect of lipids and cholesterol on the extent, size, organization and morphology of amylin aggregates was analyzed by AFM. Upper micrographs, 5 × 5 μm. At higher magnification (2.5 × 2.5 μm), single highly ordered hIAPP tetrameric and pentameric oligomeric assemblies are resolved (lower insets; scale bar, 100 nm), two of which feature a central pore (hIAPP/Lipids, 10 min, top inset). Tetrameric (lower left micrograph) and pentameric (lower right micrograph) subunits of individual hIAPP supramolecular complexes are outlined for clarity (scale bar, 50 nm). (**B**) Time-lapse 3D-AFM analysis of hIAPP aggregation on stiff mica surface. In contrast to planar membranes, self-assembled hIAPP oligomers (black arrowheads, AFM micrograph) bi-directionally extended into a mature fibril. hIAPP polymerization on mica was visualized and quantified with time-lapse AFM and 3D-section analysis, which revealed the width, length and height of full-grown hIAPP fibrils and their intermediates. (**C**) Structural diversity of hIAPP polymorphic forms on different surfaces. AFM micrographs of a single fibril, a pore and cluster self-assembled from hIAPP monomers on different surfaces are presented for clarity. AFM micrographs and fibril growth curve were adapted from References [46,84].

Similar molecular impact of lipids and cholesterol on the structure and organization of hIAPP aggregates at the membrane interface was observed in high-resolution optical and force microscopy studies. Using time-lapse AFM followed by single-particle analysis [84], the investigators reported the formation of small D = 25–35 nm spherical oligomers during the first 5 min of hIAPP addition to the incubation medium. Thereafter, hIAPP self-assembled into larger D = 90–130 nm supramolecular complexes on unsaturated negatively charged (DOPC:DOPS) anionic membranes after 10–15 min of incubation (Figure 4A, 10 min, arrowheads). On solid surfaces such as mica, hIAPP polymerized into structurally well-defined fibrils (Figure 4B; see Reference [84]). However, on lipidic DOPC:DOPS planar membranes, hIAPP oligomers did not align and elongate into fibrils, but rather assembled into channel- or pore-like supramolecular structures (Figure 4A, 10 min, arrowheads; see Reference [84]). Interestingly, AFM revealed that hIAPP oligomers preferentially accumulate on planar PC:PS membranes and much less frequently (<3% of all particles) on mica surfaces [84]. This study also revealed that hIAPP interacts with anionic membranes during the early (oligomeric) stage of aggregation, serving as a catalyst and/or template for ongoing hIAPP aggregation. At higher magnification, 3D image analysis revealed a characteristic four-fold rotational symmetry of self-assembled supramolecular complexes of hIAPP featuring a central pore (Figure 4A, hIAPP/Lipids, 10 min; top inset). Many of self-assembled hIAPP complexes on planar negatively charged lipidic PC:PS membranes exhibited tetrameric and, occasionally, pentameric globular organization (Figure 4A, bottom insets) [84].

A two-fold symmetrical organization of hIAPP and other amyloid proteins that was first incorporated into liposomes and subsequently into planar membranes has also been reported [94]. These findings bear relevance for the pathology of diabetes and other amyloid diseases, as hIAPP and other amyloid proteins interact with cellular membranes and are cytotoxic when assembled into oligomers [7,13,95]. The sizes of hIAPP globular particles assembled on planar membranes (Figure 4A; see Reference [84]) were in the same range (20–40 nm) as the soluble intermediate-sized cytotoxic hIAPP particles observed in solution [64]. In agreement with the peptide’s amphiphilic form, cytotoxic pre-fibrillar hIAPP species readily form ion-permeable channels in bilayers and in cell membranes [66,94,96,97,98]. In addition to forming channel-like structures, an AFM study showed that hIAPP also accumulates on membranes as unstructured amorphous aggregates (Figure 4A, 10 min, arrows; see Reference [84]), resembling in size (D = 300–500 nm) and morphology the amyloid deposits often associated with T2DM [99]. Incorporation of cholesterol into anionic membranes sequestered hIAPP aggregates (black arrowheads) into discrete sub-micron areas of the membrane, while protecting other regions of the membrane from hIAPP aggregation (Figure 4A, 10 min, lipids/cholesterol). Consequently, overall hIAPP capacity to form an extensive network of amyloid aggregates on the membrane was diminished in cholesterol-containing membranes [84].

Similar to other amyloid proteins, hIAPP polymerization in solution and on membranes is nucleation-dependent [7,88,92]. Consistent with the “nucleation” hypothesis, hIAPP seeding was diminished in the presence of cholesterol: a seven-fold decrease in the number of hIAPP particles was reported on planar anionic membranes that contained cholesterol as compared to those that lacked cholesterol [46,84]. This finding is consistent with the inhibitory effect of membrane cholesterol on the initial (nucleation) phase of hIAPP polymerization in solution evoked by anionic liposomes. Interestingly, AFM followed by single particle analysis revealed a large ~2-fold increase in the height of hIAPP aggregates due to this clustering effect of cholesterol [84]. The study showed that a mean volume (V_m_) of hIAPP particles was larger on lipid membranes than on lipid-free mica surface. A further, much larger increase in size of hIAPP particles was detected on PS-membranes containing cholesterol. Importantly, the authors reported a decrease in total volume (V_t_) of hIAPP aggregates on the cholesterol-containing membranes, as compared to the cholesterol-free membranes, due to a large decrease in the hIAPP seeding capacity. Overall, reconstituted studies suggest that cholesterol restricts hIAPP aggregation and accumulation on planar membranes to discrete sub-micron membrane areas, which serve as templates for the ongoing hIAPP binding and deposition (Figure 4A,C) [84].

AFM studies not only revealed polymorphism of hIAPP aggregates (Figure 4A–C), but also provided a mechanism of hIAPP self-assembly on surfaces bearing different physicochemical properties [84]. An hIAPP “growth curve” demonstrated that hIAPP monomers polymerize via two distinct mechanisms: on stiff and polar mica, hIAPP formed fibrils by longitudinal bi-directional extension of full-grown spherical oligomers, or nuclei, measuring ~6 nm in height and ~90 nm in diameter (Figure 4B); and on soft negatively charged DOPC:DOPS planar membranes hIAPP formed pore-like supramolecular structures that self-assembled out of ~25–35 nm–diameter globular subunits or oligomers (Figure 4A). AFM revealed another important feature of hIAPP aggregates on planar membranes. Amorphous deposits and channel-like structures that were formed during the early (5–10 min) stages of hIAPP aggregation on planar membranes did not transition into new structures, although the total amount and size of amorphous aggregates increased over time (Figure 4A,C) [84]. These findings signify the important contribution and long-lasting effect of lipids and cholesterol in the regulation of hIAPP aggregation.

Cholesterol is an important controller of membrane fluidity. It is found in lipid rafts of plasma and many endo-membranes, where it establishes a platform for assembly of various protein–lipid complexes important for cell signaling, endocytosis and other essential physiological processes [100]. It is possible that, by modulating membrane fluidity and/or membrane curvature [101,102], cholesterol also regulates hIAPP–lipid interactions and amylin aggregation observed in previous studies [46,66,84,98,103,104]. However, cholesterol may also directly modulate IAPP–membrane interactions. Furthermore, hIAPP monomers have a strong tendency to insert into phospholipid monolayers [105]. This could set a stage for direct peptide–cholesterol interactions in the membrane core. Bulk spectroscopy studies [46,84] support this idea: supplementation of soluble cholesterol at equimolar peptide/sterol ratio impeded hIAPP aggregation in solution in two major ways: it prolonged the lag (nucleation) phase and it decreased the fibrilization rate by 16-fold [46,84]. These results are consistent with inhibition of both fibril nucleation and elongation. These reconstituted studies have demonstrated that cholesterol may directly affect hIAPP conformational changes in solution and on membranes. Recent studies have provided additional evidence for the regulatory role of cholesterol in hIAPP aggregation and hIAPP-induced membrane damage. It is reported that cholesterol facilitates the insertion and aggregation of the N-terminal domain of hIAPP in the membrane [106]. In agreement with amylin aggregation on planar lipid membranes [84], cholesterol significantly reduced the rate of LUV-catalyzed hIAPP amyloid formation and decreased the vesicle susceptibility to hIAPP-induced leakage [104]. Fluorescence anisotropy studies demonstrated that the primary effect of the sterols on the IAPP–membrane interactions was indirect and dependent on their modulatory effect on membrane order [107]. Specific IAPP–sterol/steroid interactions had smaller effects [107]. Atomistic studies provide additional support for a major role of lipids and sterols on hIAPP aggregation on membranes. Cholesterol decreases the insertion depth of hIAPP compared to pure phospholipid membranes, while PS lipids counteract the effect of cholesterol [103]. The computational (molecular dynamics simulations) studies further revealed that this decrease in insertion depth of hIAPP in membranes is due to the increased ordering of lipids induced by cholesterol [103]. Further, hIAPP aggregation propensity strongly correlates with the insertion depth of hIAPP [103]. Thus, observed inhibitory action of cholesterol on amylin aggregation and deposition on membranes can be explained, at least in part, by its increasing ordering effect on lipid chains, thus, in turn, reducing hIAPP insertion depth and membrane seeding [46,84,104,105,108]. Consequently, cholesterol-containing membranes are less susceptible to hIAPP-induced membrane disruption and leakage [104].

In regards to potential amylin toxicity, reconstituted studies revealed a complex modulatory action of cholesterol on hIAPP-induced membrane damage [98]. The authors observed an enhancement of pore formation in raft-like DOPC/DPPC membranes and an increase in a detergent-like mechanism in POPC/POPS LUVs that contained cholesterol. This AFM study also revealed that hIAPP disrupts membranes at the boundary between the l*_o_* and l*_d_* domains [98]. Collectively, reconstituted studies suggest that both stimulatory and inhibitory effect of membrane cholesterol on amylin aggregation is possible, the propensity of which depends on membrane’s lipid composition and ordering.

## 5. Lipids and Cholesterol as Molecular Regulators of hIAPP Internalization, Aggregation and Toxicity

**Figure 5:**
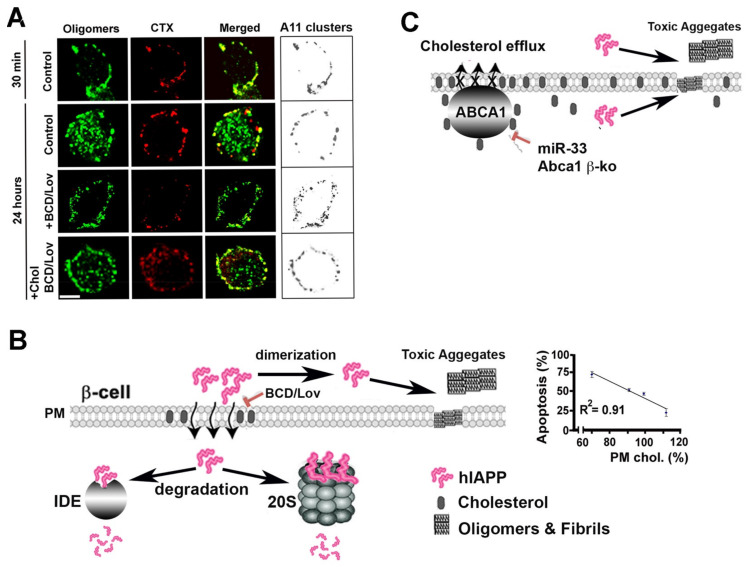
Cholesterol controls hIAPP trafficking, aggregation and toxicity in pancreatic islets. (**A**) Time-lapse laser scanning confocal microscopy (LSCM) analysis of hIAPP oligomerization and internalization in cultured human islets. Freshly dissolved hIAPP was incubated with cholera toxin (CTX) in acute human islets for indicated periods of time, in the presence or absence of cholesterol depleting agents, and the subcellular distribution, size and accumulation of A_11_-positive hIAPP oligomeric clusters was quantified by confocal microscopy and a conformation specific anti-oligomer A_11_ antibody. Bar, 5 μm. Organization and plasma membrane distribution of hIAPP oligomers (clusters) prior to and following depletion of PM cholesterol are shown on the right (boxes). LSCM micrographs adapted from Reference [66]. (**B**) Intact cholesterol organization on PM is required for internalization of hIAPP soluble oligomeric assemblies. Following internalization, hIAPP monomeric and oligomeric structures are targeted for degradation by 20S proteasome complex and intracellular proteolytic enzymes, such as insulin-degrading enzyme (IDE) [71,79]. Cholesterol depleting agents, betacyclodextrin (BCD) and lovostatin (Lov), disturb cholesterol homeostasis, leading to less hIAPP clearance and, consequently, its enhanced oligomerization and aggregation in solution and on the cell surface. Graph depicts inverse relationship between PM cholesterol content and hIAPP toxicity in human islets. Graph adapted from Reference [66]. (**C**) Disruption of cholesterol efflux in hIAPP-transgenic rodent islets stimulate hIAPP aggregation, islet amyloidosis and β-cell dysfunction. Β-cell-specific downregulation of cholesterol-specific ATP-binding cassette transporter 1 (ABCA1) was achieved by using knockout and RNAi-silencing approaches [109].

To confirm that hIAPP may also interact with native membranes, which, in turn, may affect its aggregation, and toxicity in situ, cellular and in vivo studies have been undertaken [66,108,109]. In cellular studies involving human islets, investigators systematically varied plasma membrane cholesterol levels, using cholesterol biosynthesis inhibitor lovastatin (Lov) and/or cholesterol-depleting agent, beta-cyclodextrin (BCD) and assessed the extent of hIAPP aggregation in cholesterol-containing and cholesterol-depleted cells by confocal microscopy [66]. In addition to human amylin and oligomer-specific antibodies, investigators used the lipid raft marker, cholera toxin (CTX), and the clathrin endocytotic marker, transferrin, to determine the specificity of hIAPP monomer and oligomer binding to the cell PM. Accordingly, immuno-confocal microscopy revealed a punctuated staining pattern of lipid raft marker CTX and hIAPP oligomers on the cell PM, exhibiting high spectral overlap and a high co-localization coefficients in discrete membrane regions of cultured human islets incubated with synthetic hIAPP and CTX [66]. (Figure 5A). This finding suggests that hIAPP oligomers accumulate within specific microdomains, possibly lipid rafts, on the cell PM prior to their uptake. Prolonging the incubation period from 30 min to 24 h allowed hIAPP oligomers in the microdomains to internalize, as demonstrated by a ~50% drop in hIAPP oligomer/CTX co-localization values [66]. Interestingly, upon depletion of PM cholesterol with BCD/Lov, a significant decrease in co-localization of hIAPP oligomers with cholera toxin on the cell PM was observed, indicating amylin/CTX particle de-clustering and their dispersions across the cell surface (Figure 5A). Likewise, image and single-particle analysis revealed that the mean particle area of PM-bound hIAPP oligomers in cholesterol-depleted cells decreased significantly as compared to control cells. Conversely, the number of hIAPP oligomer clusters, or puncta, on the PM of cholesterol-depleted cells increased by 3-fold relative to control cells. Consequently, cell-surface coverage by hIAPP oligomers increased by ~2-fold in cells with reduced PM cholesterol content as compared to control cells (Figure 5A, boxes). These results demonstrate that the seeding (nucleation) capacity of hIAPP oligomers and their ability to form a dense network of amyloid aggregates on the PM were augmented in cells with impaired cholesterol homeostasis. Clustering of hIAPP oligomers on the PM and their internalization by human islet cells was fully restored following replenishment of PM cholesterol (Figure 5A; see Reference [66]), indicating that hIAPP oligomer deposition on the PM is modulated by cholesterol and is reversible. Therefore, these cellular studies are in good agreement with findings of reconstituted studies showing the inhibitory and clustering effects of cholesterol on hIAPP aggregation on synthetic membranes [46,84,104].

Variations in PM cholesterol levels also seems important for hIAPP toxicity in rat and human pancreatic islet cells. Studies showed that the combination of cholesterol-depleting agents, BCD and lovostatin, reduced PM cholesterol and, in turn, increased hIAPP toxicity as compared to cells with normal membrane cholesterol content [66]. A combination of soluble cholesterol and BCD/Lov replenishes cholesterol levels comparable to controls, thus reversing the stimulatory effect of BCD/Lov on hIAPP toxicity. Thus, a strong inverse relationship between PM cholesterol levels and hIAPP toxicity in human islets was established in these studies (Figure 5B) [66]. A complementary biochemical analysis demonstrated extracellular accumulation of low-molecular-weight hIAPP monomers and dimers and intermediate-sized oligomers following PM-cholesterol depletion with BCD/Lov and their efficient clearance by islet cells upon PM cholesterol reloading with soluble cholesterol (BCD/Lov/Chol) (Figure 5B). In line with these findings, confocal microscopy shows that cholesterol supplementation stimulates, while PM cholesterol depletion decreases, hIAPP oligomer internalization and toxicity in human islet cells [66]. On the other hand, impairment of islet cholesterol transport may stimulate hIAPP aggregation and islet amyloid formation, leading to decline in beta-cell function and impaired glucose homeostasis (Figure 5C) [109]. Thus, cholesterol imparts complex regulatory effects on hIAPP turnover and toxicity in pancreatic β-cells, and these effects are of high clinical significance.

In addition to membrane cholesterol, free lipids were recently suggested as potent modulators of aggregation and toxicity of hIAPP and other amyloid proteins [110]. Previously, a theoretical model simulating the transfer kinetics of a lipid–protein complex from the aqueous phase to the lipid bilayer core has been proposed [111]. According to this model, water-soluble lipid–protein complexes penetrate the membrane faster than the bare protein provided that the hydrophobicity of the lipid–protein complex is higher than that of the bare protein [111]. Both biophysical experiments and molecular simulations carried out on hIAPP supported this hypothesis, demonstrating the key role played by free lipids in driving membrane poration mechanisms and membrane-bound fibril formation [110,111,112]. Along with these findings, studies showed that the presence of free lipids with a high critical micellar concentration (CMC), such as lipids containing short hydrocarbon chains, inhibited the hIAPP fibrilization in solution and promoted the formation of pores in LUVs [97]. By contrast, lipids with very small CMC (long acyl chains) stimulated hIAPP-induced membrane poration by the detergent-like mechanism [97]. Recently, a similar membrane-disruptive behavior was confirmed for Aβ1-40 and α-syn [110].

Based on these findings, a common molecular mechanism of membrane disruption by amyloidogenic proteins called the lipid-chaperone hypothesis was recently proposed [110]. This general model combines toxic oligomers and amyloid hypotheses in a unique framework. The lipid-chaperone hypothesis was tested on human and rat IAPP, α/β-synuclein and Aβ amyloidogenic proteins [110]. Sciacca and colleagues showed that the key players in membrane damage are the lipid–protein complexes rather than the bare proteins [110]. Specifically, the concentration of free lipids in aqueous solution acts as a switch between ion-channel-like formation, detergent-like mechanism and fibril formation in the aqueous phase. According to this model, self-assembled lipids are in chemical equilibrium with free lipids in the aqueous phase. Thus, there is a continuous exchange between self-assembled and free lipids. Following the addition of hIAPP or other intrinsically disordered proteins (IDP), a stable lipid–IDP complex is formed in the water phase. The authors provided experimental evidence that the two pathways can then occur depending on the CMC values: a high CMC value favors ion-channel-like pores, whereas a low CMC favors detergent-like mechanism. At intermediate CMC values, both mechanisms are feasible [110]. This study also showed that, in the presence of non-amyloidogenic proteins, lipid–protein complex formation is not favored, preventing protein insertion into bilayers. A study by Sciacca and colleagues provided additional evidence that oxidized lipids acting as chaperones readily form the lipid–protein complex, thus making amyloid proteins suitable to penetrate the hydrocarbon core of the bilayer [110]. Collectively, biophysical and molecular dynamics studies demonstrated that the ability of lipids to act as chaperones depends on their concentrations in solution rather than on their chemical structure [97,110,111,112]. This lipid-assisted transport mechanism proposed by Sciacca and colleagues is likely disease-relevant, given that studies show higher concentrations of linoleic acid (LA) and higher n-6/n-3 polyunsaturated fatty acid (PUFA) ratio with respect to those of healthy individuals [113]. T2DM subjects also showed impaired glycemic control which was significantly associated with unfavorable serum phospholipid n-6/n-3 PUFA ratio and greater systemic inflammation [113].

## 6. Small-Molecule Inhibitors of hIAPP Oligomerization and Fibrilization

As mentioned earlier, fully processed hIAPP has tendency to aggregate in a concentration and time-dependent manner. However, hIAPP-derived aggregates are rarely observed in non-diabetic individuals, and hIAPP’s aggregation potential in the pancreas is strongly linked with clinical manifestation of T2DM. Namely, ~90% of diabetic patients develop islet amyloidosis [6]. Thus, potent native inhibitor(s) of hIAPP oligomerization and fibrilization likely exist in human pancreatic cells and islets. One such potent inhibitor that limits hIAPP aggregation and deposition on or near cellular membranes is plasma membrane cholesterol (discussed above). The inhibitive micro-environment and constituents of β-cell granules, primarily insulin, insulin’s derived c-peptide and zinc ions (Zn), may potentially be important intracellular factors that aid hIAPP dissolution and prevent its aggregation despite high mM-storage concentrations of hIAPP and crowded microenvironment of β-cell secretory vesicles [6,114]. In vitro and computational studies revealed that c-peptide and Zn, at biologically relevant concentrations, may interact with hIAPP peptide to form stable, soluble metal–peptide complexes, thereby preventing its oligomerization and/or fibrilization [115,116]. NMR studies confirmed zinc binding to His18, and suggested zinc-induced localized disruption of the secondary structure of hIAPP in the vicinity of His18 of a putative toxic helical intermediate of IAPP [115]. In addition to c-peptide, Zn displays an ability to complex with small molecule amyloid inhibitor epigallocatechin-gallate (EGCG) and markedly enhance its anti-aggregative and anti-toxic activity against toxic forms of hIAPP [117]. Zn ions also act as an essential cofactor for the inhibitory action of insulin against hIAPP aggregation [118,119]. Similar to Zn, copper (Cu) (another essential microelement) was found to prevent hIAPP aggregation and toxicity in pancreatic β-cells by preventing its structural transitions from random coil to β-sheet-enriched oligomers and aggregates [120,121]. Metal complexes such as vanadium complexes are also potent aggregation inhibitors of the T2DM-associated hIAPP [122]. Not surprisingly, such soluble low-MW metal–hIAPP complexes showed little-to-no cellular toxicity [115,120,122]. However, under certain conditions, transitional metals or their chelators may actually accelerate hIAPP aggregation and toxicity [120,123]; this requires further investigation. Collectively, metal-based small-molecule drugs present a promising but relatively underexplored therapeutic strategy for the treatment of T2D and other amyloid diseases.

In addition to the cell’s native constituents, a number of natural products and derivates, such as small-molecule polyphenol compounds isolated from plants, showed real promise to inhibit hIAPP aggregation in vitro and in cells [124]. EGCG, a small polyphenolic antioxidant compound isolated from green tea, is a potent inhibitor of hIAPP oligomerization, fibrilization and toxicity [125,126]. EGCG interacts with hIAPP through hydrogen bonding and π–π interactions, which in turn inhibits the interpeptide interaction between hIAPP monomers and finally inhibits fibrillation of hIAPP [127]. Another natural phenol, namely resveratrol, and its derivates were particularly effective in halting hIAPP aggregation and hIAPP-induced membrane damage through screening hydrophobic interactions between hIAPP monomers and forming a stable 1:2 IAPP:resveratrol complex at the water/membrane interphase [128,129]. Similar hIAPP anti-oligomeric and anti-toxic properties were described for curcumin, a natural product found in turmeric [124]. Additionally, flavonoid rutin was shown to be protective against hIAPP aggregation and toxicity in neuronal cultures [130], expanding the therapeutic repertoire for natural products against toxic hIAPP-derived amyloids. Thus, a shared detoxifying pathway for these natural products emerged, as they all show antioxidative, anti-inflammatory and anti-aggregative actions. The molecular mechanism underlying this anti-aggregative behavior of polyphenol compounds is disruption of hydrophobic, aromatic and/or hydrogen bonds by their electron donating hydroxyl groups, which in turn destabilizes β-sheet or helical secondary structure and consequently hIAPP oligomerization and fibrilization.

## 7. hIAPP in Neurodegenerative and Metabolic Diseases: A Possible Synergy

T2DM is a progressive metabolic disease, and together with late-onset neurodegenerative disorders, such as Alzheimer’s disease (AD) and Parkinson’s disease (PD), it falls under the category of “protein misfolding diseases”. While the primary misfolded protein linked with T2DM is pancreatic hormone hIAPP, the distinctive deposition of misfolded proteins, such as tau and β-amyloid (Aβ) [131], and α-synuclein (α-Syn) has been linked with AD and PD [132], respectively. Aβ is a 36–43 aa peptide generated from the proteolytic processing of amyloid-beta precursor protein (APP) [133]. Moreover, α-Syn is a 140 aa cytosolic protein, highly enriched in the nervous tissues and, similar to APP, specifically enriched in presynaptic nerve terminals [134,135,136]. Here, we discuss a possible crosstalk and synergy between these three prominent amyloidogenic proteins.

It has been suggested that an increased level of hIAPP is a contributory but not decisive factor for pancreatic amyloid deposition and progression of diabetes [137]. Similarly, using β-cell-specific human IAPP transgenic mice, Singh and colleagues showed a significant acceleration of severe diabetic symptoms and accumulation of Th-S-positive amyloid-laden plaques in the pancreas of aged mice compared to the younger littermates despite comparable hIAPP protein levels [33], indicating contribution of co-precipitation factors in hIAPP aggregation and likely islet amyloidosis in T2D subjects. Interestingly, hIAPP toxic oligomers were found in the brains of hIAPP transgenic mouse, and an IAPP-receptor channel blocker was effective against hIAPP toxicity in neuronal cultures, suggesting its possible role in neuron demise associated with brain proteinopathies [138,139]. Toxic human amylin oligomers and aggregates also accumulate in hIAPP transgenic Drosophila melanogaster and C. Elegans models [140,141]. Importantly, pan-neuronal expression of hIAPP in the nervous system of C. Elegans induced diverse transcriptional changes and pathophysiological responses in various signaling pathways [142]. Consequently, a specific defect in neural developmental programs, as well as sensory behavior in C. Elegans animals expressing human amylin, was reported [142]. In support of hIAPP neurotoxicity, hIAPP-derived protein aggregates have been recently discovered in brain plaques of T2DM patients exhibiting dementia [139,143].

Along this notion, several epidemiological studies have insinuated crosstalk between brain and islet amyloid proteins, as well as a common ethological link between T2DM and neurodegenerative diseases, such as AD and PD [144,145,146,147]. Additionally, it is becoming increasingly clear that both hIAPP and Aβ share similar aggregation and toxic features, thus indicating common pathogenic mechanisms (reviewed in Reference [148]). Recently, Martinez-Valbuena and colleagues, using 138 subjects with neurodegenerative diseases or T2D, demonstrated increased immunoreactivity of IAPP, α-Syn, tau and prion protein in pancreatic cells of these subjects, as compared to controls. This indicates the possible contributory roles of these proteins toward the complex pathophysiology of T2DM and in the development of insulin resistance in AD and PD [145]. Although the detailed mechanism is not clear, cross-seeding between IAPP and Aβ/α-Syn has been proposed as the link [137]. To verify this cross-seeding mechanism, Wang and Westermark (2021) used bimolecular fluorescence complementation (BiFC) assays and *Drosophila melanogaster* expressing hIAPP/Aβ to show heterologous interactions between hIAPP and Aβ, and co-deposition of these amyloid-forming peptides in animal brains, resulting in reduced fly longevity [137]. Similarly, using biophysical approaches, Horvath and colleagues showed that the interactions between hIAPP and α-Syn accelerated their aggregation in vitro [149]. From these reconstituted and in vivo studies, it became clear that the presence of hIAPP can stimulate α-Syn and Aβ-aggregation and possibly amyloid plaque formation in the brain. This observation may explain why T2DM patients are susceptible to developing PD and AD [144,145,146,147]. Interestingly, a recent study identified α-Syn as a component of islet amyloid extracted from human IAPP transgenic mice and T2D individuals [150]. Accelerated β-cell amyloid formation in human IAPP transgenic mice following tail-vein injection of αSyn/Snca −/− (α-Syn encoding gene) background further supported a role for α-Syn in pancreatic β-cell amyloid formation [150].

It has been reported that α-Syn strains are determined by species of misfolded seeds, intracellular environments and protein–protein interactions [151,152]. Accordingly, the study by Martinez-Valbuena et al. (2021) demonstrated the presence of different forms of α-Syn in pancreatic β-cells of subjects with neurodegenerative diseases or T2D [145], indicating the existence of a complex network of cross-reactivity between islet and brain amyloid proteins. Mechanistically, it has been proposed that the initial formation of misfolded α-Syn occurs in the gut and then spreads to the brain via peripheral autonomic nerves, thus affecting several other organs, including the heart and intestine [153]. A recent study by the Van Den Berge group, using rats as an experimental model system, demonstrated marked age-dependent gut-to-brain and brain-to-gut spreading of α-Syn pathology along the sympathetic and parasympathetic nerves, and age-dependent dysfunction of the heart and stomach, as in patients with Parkinson’s disease [154]. Accordingly, it has been reported that trans-neuronal propagation of α-Syn pathology leads to sensory neuron dysfunction and neuropathic impairment, suggesting one of the pathogenic mechanisms underlying PD [155]. Likewise, it has been hypothesized that the initial formation of hIAPP aggregate occurs in the membrane enriched compartment within the β-cell and is eventually transmitted to the extracellular space [6]. Although the underlying mechanism is not clear yet, a study using transgenic mouse models and T2DM human tissue showed the prion-like spreading mechanism of IAPP [156]. Further biochemical and cell-biology studies are necessary to confirm this putative cross-seeding link between brain and islet amyloid proteins. In particular, isolation of pancreatic hIAPP-induced α-Syn and Aβ cross-seed conformers and their direct neuronal/β-cell toxicities would provide further evidence and conceptual grounds for the pathological role of hIAPP in α-Syn and Aβ peripheral aggregation and subsequent prion-like spreading.

## 8. Conclusions

In summary, molecular and cellular studies spanning the last two decades provided novel mechanistic insights into structure, function and toxicity of amyloid proteins, such as hIAPP. Studies have revealed that natural polyphenolic products and essential cellular constituents, such as metals, membrane-associated lipids and cholesterol, potently modulate aggregation and cytotoxicity of hIAPP, and this, in turn, may affect broad cellular and systems functions, such as glucose homeostasis. Besides unravelling the important roles of metal–amyloid and lipid–amyloid complexes in the etiology of amyloid diseases, new emerging evidence points to synergistic actions, interactions and cross-seeding of hIAPP and other amyloid proteins at various locations in human body, most notably the brain and the pancreas. An intriguing idea that awaits verification is that these co-precipitation factors may work together to worsen pathology and clinical outcome of amyloid-laden tissues and organs. In addition to natural products, cholesterol-modifying and metal-chelating agents, the use of synthetic small-molecule activators and inhibitors of cell proteolytic system, proteasome, lysosome and soluble or membrane-associated proteolytic enzymes offers exciting opportunities for the development of novel approaches and drugs that may halt or slow down the progression of amyloid-associated diseases.

## 9. Future Directions

In spite of the significant progress and conceptual advancements in regards to general mechanisms of hIAPP aggregation and toxicity, there is still a gap in understanding cellular pathways and mechanisms that prevent hIAPP from aggregating and forming amyloid in cells and organs. Particularly challenging and lacking are studies that will identify transient cytotoxic hIAPP structural intermediates in vivo and the inherent molecular mechanisms involved in cell/tissue protection. To capture formation and recycling/degradation of hIAPP transient toxic and non-toxic molecular species, a real-time single-molecule resolution technique that is compatible with intact cellular analysis will be required. Emerging evidence of the important inhibitory role of natural products and essential cellular constituents opens opportunities for early targeted intervention during hIAPP-induced islet amyloidosis and possibly other amyloid diseases. Finally, recent technological advancements in computational biology, structural biology and synthetic chemistry have opened a new frontier and opportunities for designing novel small-molecule drugs that will selectively and efficiently stimulate cellular process and pathways involved in hIAPP clearance and detoxification in pancreatic and other tissues. This gain-of-function approach can be utilized alone or in conjunction with amyloid inhibitors designed to directly target and disrupt cytotoxic amyloid aggregates.
